# Supplementary material for: Are carbon emissions trading and green financial instruments synergistic? -Comprehensive quantitative research based on content analysis
Source: PLoS One. 2024 Mar 7;19(3):e0298601. doi: 10.1371/journal.pone.0298601 (PMC10919601; doi:10.1371/journal.pone.0298601)
Supplement: S1 Data — (ZIP) [file pone.0298601.s001.zip › Data/地级市Z_F工作报告.zip!/╜¡╦╒/┴1⁄4╘╞╕█╩╨/└┤╫╘╣1⁄2╓┌║┼í╛┬φ┐╦╩2╛▌═°í┐_2011.docx]

各位代表：

现在，我代表市人民政府向大会作工作报告，同时根据中共连云港市委十二五规划的建议，市政府制定了《连云港市国民经济和社会发展第十二个五年规划纲要》草案，请一并予以审议，并请各位政协委员提出意见。

十一五时期和2010年工作回顾

十一五时期是我市发展史上极不平凡的时期，是创造重大机遇、经受重大考验、取得重大成就的时期。在中共连云港市委的领导下，全市上下深入贯彻落实科学发展观，积极应对复杂多变的宏观经济形势，凝心聚力、开拓进取、创业创新，谱写了科学发展、跨越发展的历史新篇章。

十一五的五年，是致力推动跨越崛起、不断开创发展新局面、综合实力显著提升的五年。

区域发展格局高起点构建。坚持高端定位和谋划，成功推动以连云港为龙头的江苏沿海地区发展规划获得国家批准。重视规划引领作用，编制东部滨海地区发展战略规划，完成新一轮城市和港口总体规划修编，制定专项规划140余项，构筑了一心三极国际性海滨城市、一体两翼组合大港、一纵一横产业走廊的发展布局。加快市临港新兴产业区建设，推进柘汪、板桥、燕尾、灌河等沿海产业园区和沿东陇海线都市工业带产业集聚，大规模启动徐圩新区开发，生产力布局不断优化。连云港在全国、全省发展大局中战略地位进一步凸显。

国际金融危机冲击有效应对。面对历史罕见的国际金融危机带来的严峻挑战，出台稳定工业生产、扶持中小企业、促进外贸稳定系列工作措施，落实扩大内需政策，加大投资和项目建设力度。兑现税费减免政策，办理减免各项税费20亿元、出口退税24.8亿元。加强财政金融支持，设立创业投资引导、国际市场开拓等专项资金2亿元，为5000多户中小企业担保各类贷款总额100多亿元，有效保障了经济稳定发展。

工业化城市化快速推进。累计完成工业投资2500亿元，是十五时期的4倍；规模以上工业总产值达1941亿元，是十五末的5倍，工业利税年均增长40%。三次产业结构由十五末的20.5:44.2:35.3调整为15.3:47.4:37.3。中心城市建成区面积扩大到120平方公里、人口100万，区域性航运、商务、金融、信息、物流、旅游六大中心启动建设，四县县城加速向中等城市迈进。全市城市化率达45%，较十五末提高6.7个百分点，二三产业从业人员比重提高11.9个百分点。

经济综合实力不断增强。五年来，地区生产总值年均增长14.2%，总量翻了一番多。财政总收入、一般预算收入年均增长44.3%、41.9%，总量均翻两番以上。完成全社会固定资产投资4000亿元，是十五期间的3.4倍。金融机构存款余额达1243.8亿元、贷款余额946.3亿元，是十五末的2.8倍和3倍。中心城市入选中国投资环境百佳城市和《福布斯》大陆最佳商业城市榜。四县经济总量全部实现翻番，市开发区在54个国家级开发区综合评价中位次前移10位。

十一五的五年，是着力突破重大事项、展开新一轮沿海开发、区域发展后劲显著增强的五年。

港口服务功能大幅提升。完成港口建设投入190亿元，是十五期间的5.8倍。主体港区15万吨级航道建成通航，30万吨级航道先导试挖工程完成阶段性目标，30万吨级矿石码头试运营，12个生产性泊位建成投产。赣榆港区防波堤开工建设，灌河口航道整治有序展开，燕尾港3万吨级散货码头加快实施。港口新增吞吐能力5400万吨、集装箱200万标箱。集疏运体系加快完善，基本建成南疏港、东疏港通道和内河港中云台作业区一期工程。新辟内外贸航线40条，设立内陆无水港8个，新亚欧大陆桥过境集装箱运输西延至北欧。口岸公共信息平台功能不断完善。港口带动沿海开发的龙头作用和服务区域发展能力不断增强。

基础设施建设成效突出。市域高速公路骨架路网基本形成，新增通车里程112公里，连盐高速公路、连临高速公路连云港段建成通车，新建改建国省干线公路563公里，临海高等级公路加快推进，完成东陇海铁路电气化改造，开工建设连盐铁路，建成使用民航机场国际候机厅。在全省率先完成海堤防护达标任务，建成新沂河整治、大浦河调尾、盐灌船闸等工程，城市防洪减灾和水资源供给能力得到提升。新建高压变电所37座，地区电网新增规模较十五期间翻一番。完成市区有线数字电视整体转换，互联网普及率较十五末提高20个百分点。区域发展承载功能进一步提升。

重大产业项目集聚发展。五年新上投资亿元以上工业项目400多个，过10亿元项目28个。田湾核电一期工程投入运行，市开发区建成中海集装箱、国电联合动力等装备制造项目，风电装备形成完整产业链。徐圩新区、板桥园区珠江钢管、环球铜业、甲醇制烯烃、镍合金制造、益海化工等基地型大项目成功落户。北翼新海石化等一批临港产业项目建成投产，南翼灌河口船舶产业集聚地初步形成。沿东陇海线产业带太阳雨太阳能、晶海洋半导体等光热光伏产业快速发展。重大项目储备后劲增强，超超临界机组、炼化一体化、精品钢铁等一大批项目前期工作扎实推进。

十一五的五年，是坚持创新驱动、加快结构调整、转型发展取得显著进展的五年。

科技创新深入实施。与20多家高校院所签署科技合作协议，校企联盟发展到330个，共建研发机构120家，中科院能源动力研究中心、省海洋资源开发研究院形成研发能力，南京大学高新技术研究院、南京工业大学海洋工程研究院等一批创新载体加快建设。全市企业院士工作站达4家，拥有国家和省级企业博士后工作站24家、工程技术中心26家，硅产品、高性能纤维和风力叶片3个检测中心加快建设。承担国家重大科技专项19项，新上火炬、星火等国家计划234项，获国家和省部级科技进步奖20项。全市科技孵化器面积达12万平方米，研发投入占地区生产总值比重1.3%，科技进步贡献率40%，较十五末提高0.46和6.3个百分点，跻身全国科技进步先进市行列。建立高层次人才引进专项资金，实施创业创新领军人才集聚555工程，全市人才保有量年均增长11%，有力支撑了区域创新能力提升。

先进制造业快速成长。实施高新技术产业双倍增计划，创建4个国家级高新技术产业化基地，6个特色产业基地建设进入省双百工程，4家医药企业入选中国制药工业百强，万吨级碳纤维基地一期工程建成投产，获批国家重点新产品、省高新技术产品225个，高新技术产业产值翻三番。工业化与信息化融合发展，获批省制造业信息化科技示范市。新型临港基础产业培育力度加大，产值占工业产值比重39%，较十五末提高10个百分点。专利、标准和品牌战略深入实施，专利授权量年均增长46%，创建中国驰名商标3件，企业品牌竞争力不断增强。

服务业加快发展。港口物流快速增长，吞吐量迈入亿吨大港行列、集装箱运输进入全国十强，年均增长17.6%和30.8%，分别翻了一番多和两番。民航年客运量达42.3万人次，是十五末4.3倍。现代商贸流通业态快速发展，社会消费品零售总额年均增长19.2%。举办国际西游记文化节、江苏沿海旅游节等节庆活动，新增国家AAAA级景区6个，游客接待量、旅游收入年均分别增长15%、18%。完成房地产开发630万平方米，商品房销售面积年均增长53.9%。区域性国际商务中心初具规模，商务、会展、信息等新兴服务业蓬勃兴起，市开发区成为省级服务外包示范区。服务业增加值年均增长15.3%，比十五期间提高3.7个百分点。

现代农业提档升级。高效农业、设施农业规模扩张，面积分别达210万和85万亩，占耕地比重37.5%和15.2%。建设国家级粮食万亩高产示范项目20个，粮食生产连续八年丰产丰收，东海县被评为全国粮食生产先进县标兵。农业园区化发展快速推进，建成市级以上现代农业产业园17家，赣榆海水工厂化养殖、东海鲜切花、灌云设施蔬菜、灌南食用菌等特色产业呈现规模化集聚。农业产业化经营加快推进，培育市级以上龙头企业166家。农产品质量安全建设不断加强，认定无公害农产品产地面积420万亩，创建农畜产品品牌470个。农业外向化水平提高，利用外资领先苏北，农产品出口居全省前列。

资源环境保护持续加强。坚持集约节约利用土地资源，落实最严格土地管理制度，清理收回闲置建设用地8700亩，复垦开发农用地18.8万亩。坚持科学用海、合理开发，海域管理不断加强。强力推进节能减排，关停化工生产企业80家，实施150项节能技改项目，建成8个污水处理设施和6处集中供热设施，单位地区生产总值能耗下降20%，化学需氧量和二氧化硫减排完成省定目标，淮河流域治理工程达到国家考核要求。完成绿化造林103万亩，森林覆盖率达22.2%，较十五末提高7.1个百分点，四县建成国家级生态示范区。

十一五的五年，是深化改革开放、加速资源要素集聚、发展活力显著提升的五年。

各项改革向纵深推进。实施市区行政区划调整，完成新一轮政府机构改革，组建徐圩新区管委会，推进云台山景区和科教创业园一体化发展。完善市对区财政管理体制。完成市属国有企业改制扫尾，妥善安置破产企业职工和离退休人员8400余人，实现经营型事业单位改制转企，建立公益型事业单位岗位管理和绩效工资制度。医药卫生体制五项重点改革扎实推进，开展基本药物制度试点。加快投融资体制创新，成立市级国有资产经营平台4家，实现港口股份上市融资，发行企业债券48.5亿元，创立中科黄海股权基金。城市商业银行重组并入江苏银行，完成3家农村信用联社改制，组建市县邮政储蓄银行、东海村镇银行，引进外地5家农商行设立分支机构，建立8家农村小额贷款公司。农村综合改革扎实推进，开展政策性农业保险，完成集体林权制度改革，实现土地流转119.9万亩，发展农民合作组织2289家，带动农户58.6万户。东海县安峰镇列入省级强镇扩权改革试点。

开放型经济成效明显。深入实施开放兴市战略，组织开展沿海开发系列推介，连续举办苏北投资贸易洽谈会等大型经贸活动，累计新批外商投资项目833个，实际到帐注册外资40亿美元，是十五期间的2.4倍，内联客方到位资金1600亿元，年均增长23.8%。实现外贸进出口总额189亿美元，年均增长19%；完成出口99亿美元，是十五期间的3倍。国际服务贸易年均增长28.7%。有效规范出国劳务市场，完成外经营业额9亿美元。加快优势企业走出去步伐，新批境外投资项目37个，是十五期间的6.4倍。各类开放载体功能不断完善，带动能力提高。出口加工区二期建成运营并实现保税功能叠加，保税物流中心封关运作，化学工业园和连云、海州、灌云经济开发区升格为省级开发区，新增南北共建园区5个、省级特色产业园4个。10个综合类开发区注册外资实际到帐38.3亿美元，年均增长38.8%。

十一五的五年，是积极打造国际性海滨城市、扎实推进新农村建设、城乡一体化显著加快的五年。

特大城市框架全面拉开。大力推进城市建设重心东移，实施重点城建项目3600项，累计投入资金960亿元，是十五期间的3倍。建成花果山大道城市主轴和海宁大道、东方大道、北崮山大道等骨干路网，打通242省道，城市空间布局优化，拉开了三极互动的特大城市框架。连云新城10平方公里示范区基础设施启动建设，起步区海州湾商务中心、胜利湖等重点工程建成，东哨西墅片区初具形象。科教创业园路网框架基本形成，高校科研区、公共体育区初具规模。孔望山新城、凤凰新城开发加快推进。东部城区形象加速刷新，中央商务区建设、连岛开发加快推进，在海一方公园、鑫港大厦、金海商务大厦等一批标志性建筑建成，海棠路立交、铁路连云港东站投入使用，山海景观轴、滨海休闲带有序推进，海滨特色风貌初步展现。

城市功能不断增强。强化公用设施及配套建设，新建改建主次干道130条，发行公交IC卡，新增优化公交线路40条，开通新浦至连云地区公交车，新增自来水管网380公里、集中供热250万平方米，发展管道天然气用户5万户，燃气普及率98%，城市污水处理率达80.7%。建成民防应急指挥中心，新增人防工程59万平方米。实施旧城改造项目260个，拆迁改造面积600多万平方米。加强居住小区物业管理，新增省级优秀物业管理小区26家。建成青年公园、东盐河风光带等一批景观工程，市区绿地率达37.1%。有效开展集中式饮用水源地和重大污染隐患治理，建成大圣湖应急水源。开展城市环境综合整治，增加垃圾转运站28座，依法拆除违法违章建筑45.5万平方米，完成美化亮化面积85万平方米，市区环境质量逐年改善，空气质量优良率91.4%，公众环境满意率达85.6%。国家环保模范城创建通过技术评估、卫生城创建扎实推进，成功创建省级文明城市、园林城市，城市人居环境持续改善。

新型城镇化加快推进。坚持县当城建、镇当城建，实现村镇规划全覆盖。灌云新城展现良好形象，灌南县城规模扩大，赣榆新城核心区有序展开，东海县城湖滨特色逐步显现。创建省、市级城市化示范镇和新型小城镇79个，东海温泉镇创成省级园林小城镇，赣榆海头镇成为国家发展改革试点镇，灌云燕尾新城初具规模，灌南半岛新区开发全面铺开。实施农村改革发展六大行动计划，扎实兴办新一轮实事。新建改建农村公路8200多公里，解决136万人饮水安全问题，行政村客运班车通达率99.4%，完成中低产田改造93.2万亩，农业机械化率达72.5%。完成400多个村庄整治，疏浚县乡河道900多条，发展沼气用户12万户，新增无害化卫生厕所19.7万个。加强康居示范村建设，新农村服务中心村级全覆盖，食品、日用消费品、农资乡级农家店覆盖面100%。农村生产生活条件不断改善、面貌明显改变。

十一五的五年，是加强社会建设、增进民生福祉、城乡群众生活显著改善的五年。

人民生活水平不断提高。落实积极的就业政策，加强各类职业技能培训，新增就业33.9万人，转移农村劳动力28.5万人，实现下岗失业人员再就业9.9万人，零就业家庭动态为零。推动以创业带就业，大力发展民营经济，新增私营企业1.4万家、注册个体工商户13.9万户。组织百名教授科教兴百村等支农活动，有效带动农民增收。城市居民人均可支配收入、农民人均纯收入年均增长13.6%、12.1%。居民消费结构优化，价格水平总体平稳，教育文化、休闲娱乐和健康消费比重提高。城市每百户家庭电脑、移动电话拥有量较十五末分别增长135%、182%，汽车拥有量5.5辆。城镇和农村人均住房面积分别达35.4、33.5平方米，较十五末增加3.8和9平方米。城乡居民恩格尔系数较十五末降低4.6个百分点。

社会保障进一步加强。企业职工养老保险覆盖面达95.2%，新型农村养老保险、合作医疗参加率达98%和99.5%，工伤、生育保险覆盖面均达95%，在职职工参加失业保险人数29.6万人，基本实现城乡居民医疗保险全覆盖，被征地农民基本生活保障实现即征即保。完善住房保障体系，为1.3万户职工家庭发放住房公积金贷款56亿元，累计投资24亿元加快保障性住房建设，开工建设经济适用房1.8万套，筹集、建设廉租住房1791套，2.8万户中低收入家庭解决了住房困难，城市低收入无房家庭基本实现应保尽保。完善社会救助体系，健全困难职工帮扶网络，建立城乡低保标准自然增长机制，实现城乡低保全覆盖，特困群众基本生活得到保障。农村五保对象集中供养率达70%。实施脱贫攻坚工程，帮助37万农村贫困人口脱贫解困。红十字博爱系列活动广泛开展，慈善事业、光彩事业得到新发展。

社会事业全面进步。公共财政用于社会事业支出累计达223.6亿元，是十五期间的3.9倍。市文化艺术中心、博物馆、体育馆、急救中心、传染病医院、二院东院区、一院综合楼、中医院门诊楼、妇女儿童活动中心、气象防灾减灾指挥中心等一大批社会事业项目投入使用或基本建成。城乡办学条件和教育质量进一步改善，中小学校舍安全工程扎实推进，市区新建成5所中小学校。全面落实义务教育免费政策，基本普及15年基础教育，小学、初中入学率均达100%，高中段毛入学率96.9%。创建省级以上示范性职业学校10所，职业教育服务发展的能力得到提高。师范专科学校、职业技术学院等4所院校完成迁建，淮海工学院办学层次提升。公共卫生体系逐步健全，新增三甲医院3所，城市社区和农村三级医疗卫生服务体系全覆盖，城乡15分钟健康服务圈基本形成。广播电视、文艺创作、文物保护进一步加强，有线电视入户率80%。组织创作演出《大潮连云》、《梦境西游》等优秀剧目，和谐文化进万家被评为全国特色广场文化活动，新增省级以上文物保护单位7处，3个项目入选国家非物质遗产保护名录。实现人口计生服务体系全覆盖，人口自然增长率6.9‰。在苏北率先成为省体育强市，全民基本健身设施城乡全覆盖，举办国际和全国性赛事13项，在省十七届运动会等省级以上体育竞赛中获得金牌148枚。

社会管理不断加强。平安连云港建设深入推进，社会治安综合治理全面加强，科技强警示范市建设通过国家验收，群众治安满意率为97%。灌南县被评为全国平安建设先进县。认真落实安全生产责任制，强化基层基础工作，加大执法管理力度，安全生产形势保持平稳。完善食品药品安全综合监督体系，开展农资、食品、药品、乳制品等专项治理，市场秩序进一步规范。突发事件应急预案体系逐步完善，应急救援队伍建设有效加强。建立12345政府服务热线，四级便民服务网络不断健全。完成第二次经济普查、第六次人口普查。侨务、外事、对台工作有效服务经济建设，防震减灾、老区开发、档案、地方志、残疾人、老龄、关心下一代事业不断发展。

精神文明和民主法制建设成效突出。群众性精神文明创建蓬勃开展，12家单位被评为全国文明单位和文明村镇，690余人被评为市级以上劳动模范和市各行各业首席员工，获全国未成年人思想道德建设工作先进市称号，社会文明程度不断提高。完成对口援建四川地震灾区任务，积极开展援疆工作。法治连云港建设有序推进，完成五五普法工作。加强政务公开，提高行政效能，清理精简审批事项42项，建成行政权力网上公开透明运行系统。廉政建设扎实推进。加强行政监察和审计监督。认真听取人大代表、政协委员意见和建议，自觉向市人大常委会报告工作，向市政协通报情况，累计办理人大代表建议732件、政协委员提案1824件。村务公开和基层民主政治建设有效推进，村委会依法自治达标率97%。建立大调解机制，实施信访工作基层基础建设提升工程，有效化解社会矛盾。民族宗教、国防建设、民兵预备役和优抚安置工作得到加强，实现全国双拥模范城五连冠。

刚刚过去的2010年，全市经济社会各项工作取得了新的成绩，为十一五发展划上圆满句号。这一年，经济总量跨上新的台阶。预计完成地区生产总值1150亿元，同比增长14%；实现财政总收入352.6亿元、一般预算收入141.4亿元，分别增长51.8%、56.7%；全社会固定资产投资1234.2亿元，增长23.4%；工业增加值、服务业增加值、社会消费品零售总额均超过400亿元。这一年，沿海开发三年行动初战告捷。新开工和在建亿元以上项目210个，中彩多晶硅、东茂矿业、风力发电等一批临港产业项目建成投产，两家企业产值超过百亿元，通榆运河通水通航，空港实现临时对外开放，完成港口吞吐量1.35亿吨、集装箱运量387万标箱。这一年，创新型经济蓬勃发展。编制创新型城市发展规划，国家重点实验室、省创新药物研究院等6个重大创新载体立项建设，清洁能源产业园入选省级创新园区，创建省创新型企业20家，康缘药业成为全国创新型试点企业，5兆瓦风电叶片等创新产品成功下线，高新技术产业产值达595亿元，增长48.4%。被中央确定为全国学习实践科学发展观、转变发展方式有特色的五个典型城市之一。这一年，支撑跨越发展的资源要素加快汇聚。与郑州、西安等陆桥沿线重要节点城市合作建设大陆桥物流联盟，成功举办中日新丝绸之路企业合作论坛、江苏民营企业家高层论坛。引进注册外资实际到帐11亿美元，内联客方到位资金435亿元，完成出口26亿美元，增长33.1%。发行企业债券23.5亿元、公司债6.5亿元，吸引异地金融机构投放贷款余额130亿元。引进各类人才7000余人。这一年，城乡群众生活得到新改善。城镇登记失业率控制在3%以内，城市居民人均可支配收入19000元、农民人均纯收入6850元，均增长12%，企业退休人员基本养老金人均增长10%以上，实现与上海异地就医结算，五项社会保险参保率均超过95%。新农合、城镇居民医保筹资水平和低保标准进一步提高，居民消费价格指数低于省控标准。

回顾十一五的工作，我们不仅取得了超出预期的发展成果、创造了连云港发展的一个黄金期，而且提高了在经历考验中砥砺奋进、在创新思路中破解难题的能力，积累了在复杂环境下加快发展、在后发情况下推动跨越的宝贵经验。这就是：必须坚持高端定位，引领发展；坚持城乡统筹，协调推进；坚持重点突破，以点带面；坚持创新发展，激发活力；坚持以人为本，利民惠民。成绩来之不易，经验弥足珍贵。这是中央和省委、省政府正确决策、关心支持的结果，是全市人民在市委坚强领导下团结一致、艰苦奋斗的结果，是市人大、市政协加强监督、鼎力支持的结果。我代表市人民政府，向全市人民，向人大代表、政协委员，向各民主党派、工商联、人民团体和社会各界人士，向驻连部队、武警官兵、公安干警，向为连云港改革发展稳定作出贡献的驻连部省属单位，向所有关心支持连云港建设的海内外朋友，表示衷心的感谢和崇高的敬意！

在总结成绩的同时，我们清醒看到，我市发展中还存在许多困难和矛盾，政府工作也存在一些不足和差距。主要表现为：经济总量仍然偏小，结构还不尽合理，发展的资源环境约束加剧；科技进步和创新的引领支撑作用有待进一步增强，现代产业体系需要加快构建；新型城镇化水平还不高，基本公共服务体系需要进一步完善；就业仍然面临较大压力，保障和改善民生的任务还相当繁重；机关服务软环境还需要进一步改善。对此，我们将进一步增强忧患意识和责任意识，采取更加有力措施，切实加以解决。

十二五时期目标任务

各位代表，党的十七届五中全会科学判断当前和今后一个时期仍处于可以大有作为的重要战略机遇期。展望未来，今后五年正是连云港推进沿海开发的关键期、建成全面小康的攻坚期、奋进后发先至的加速期。世界经济调整复苏的趋势仍将继续，国内经济社会发展长期向好的态势仍将持续，我市多重叠加机遇的带动效应仍将延续，特别是十一五发展取得的巨大成就，为在新的起点上加快全面跨越奠定了坚实基础。尽管还面临宏观环境复杂多变、区域竞争日益激烈、转型发展困难交织的压力和挑战，只要我们始终保持昂扬奋进的豪气、强力推进沿海开发，保持浴火重生的勇气、加快转变发展方式，保持开拓创新的锐气、矢志推动跨越崛起，就一定能够夺取十二五发展的新胜利！

根据市委十届十次全会部署，全市十二五发展的指导思想是：坚持以邓小平理论和三个代表重要思想为指导，深入贯彻落实科学发展观，积极抢抓江苏沿海开发和长三角一体化机遇，以推动科学发展、建设幸福港城为主题，以转变发展方式、推进转型升级为主线，以改善人民生活、促进社会和谐为根本，以深化改革开放、推动创新发展为动力，认真落实改革发展稳定各项措施，加快实施港口龙头牵引、新型产业培育、基础设施支撑、城市功能提升、城乡统筹发展、社会和谐构建工程，统筹推进经济社会事业发展，以县为单位全面建成小康社会，加快推进后发先至，为基本实现现代化打下更加坚实基础。主要奋斗目标是：

--综合实力再上新台阶。到十二五末，地区生产总值和人均地区生产总值实现翻番，财政总收入和一般预算收入均翻一番以上；累计固定资产投资超过万亿元，实际利用外资冲刺百亿美元。发展速度在全省争先，经济总量在全国沿海进位。四县力争进入全国百强县行列。

--沿海开发取得新突破。完善以港口为龙头的基础设施体系，建成30万吨级航道、30万吨原油码头等一批重大工程，徐圩新区港产城一体化建设取得重大进展，赣榆、燕尾、灌河港区和空港实现对外开放，保税港区、国家东中西区域合作示范区加快建设，港口吞吐量达2.5亿吨和800万标箱，基本实现再翻番。大力发展高新技术产业、临港基础产业，培育七大千亿产业集群，初步形成现代化的新型临港产业基地。

--创新发展实现新跨越。充分发挥科技第一生产力和人才第一资源作用，加快推进发展方式转变。构建以企业为主体的技术创新体系，建成国家清洁能源科技创新示范基地、半导体产业集聚标准化示范区、创新药物和新材料研发与产业化示范基地，打造一批创新型企业和园区，争创国家级高新区，进入国家创新型城市试点。研发投入占地区生产总值比重提高到2%，科技进步贡献率提高到50%，争取主要科技创新指标接近或达到国内先进水平。

--城市建设彰显新形象。区域性航运、商务、金融、信息、物流、旅游六大中心建设取得阶段性成果。连云新城、科教创业园、徐圩新区等重点片区开发快速突破，一心三极城市功能区融合发展，海滨特色风貌进一步展现。中心城市建成区面积达150平方公里、人口150万。新型城镇化加快推进，特色鲜明、功能互补的区域城镇体系基本形成，城市化率达56%。基本建成面向东北亚、沟通中西部的区域性特大型国际海港中心城市。

--生态文明得到新提升。积极推进绿色发展，倡导使用低碳技术，大力发展循环经济，致力建设国家循环经济示范区，申报国家生态工业示范园。持续加强节能减排、污染治理、环境保护，单位地区生产总值能耗下降和主要污染物排放量削减达到国家控制要求，推动资源节约型、环境友好型城市建设。加强生态功能区建设，城市绿化覆盖率达43%，森林覆盖率提高到24.5%，建成国家园林城市、卫生城市、环保模范城市，争创生态城市。

--和谐构建开创新局面。坚持统筹城乡一体化发展，逐步缩小城乡差距。促进社会就业更加充分，推动居民收入与经济发展同步增长，力争城乡居民收入六年翻番。大力发展社会事业，逐步完善覆盖城乡居民的终身教育体系、公共服务体系、社会保障体系。加快建设社会主义核心价值体系，提高全社会文明程度，争创全国文明城市，提升城市文化软实力。完善社会管理机制，深入推进法治连云港、平安连云港建设，成为全国、全省最安全地区之一，不断提高港城人民的幸福指数。

2011年主要工作

2011年是中国共产党成立90周年，是十二五发展的开局之年，做好今年的工作至关重要。根据中央、省经济工作会议和市委十届十一次全会精神，今年全市经济社会发展主要预期目标是：地区生产总值增长13%，完成全社会固定资产投资1400亿元，财政一般预算收入增长20%，城市居民人均可支配收入、农民人均纯收入均增长12%，城镇登记失业率在3.6%以内，居民消费价格水平不高于省控标准，节能减排完成省定任务。围绕实现上述目标，着重做好七方面工作：

一、保持经济强劲增势，扎实开启五年发展新征程

突出需求拉动，强化运行调度，着力巩固经济持续快速增长态势，确保十二五发展开好局、起好步。

强化投资拉动作用。坚持扩大有效投入，有序启动十二五规划重大项目建设，引导资金投向产业发展、重点片区开发、基础设施建设、社会事业、民生保障、生态环境等重点领域。集中力量实施一批市级重点投资项目，增强经济增长拉动力。开展工业项目三百工程大推进活动，建设投资亿元以上的重点开工项目100项、重点竣工项目100项和投资3000万元以上的重点技术改造项目100项，工业投资达到950亿元，规模以上工业增加值增长17%。实现建筑业产值增长15%以上。落实鼓励引导民间投资的政策措施，提高民间投资在社会投资中的比重，促进民营经济加快发展。

积极扩大消费需求。落实国家鼓励消费的政策，改善消费环境，稳定消费预期，提高居民消费能力，社会消费品零售总额增长18%。着力培育消费热点，抓好家电、汽车、摩托车下乡和以旧换新工作，拓展文化娱乐、休闲健身、信息通讯等多样化服务消费，促进住房消费健康发展，实现商品房销售面积550万平方米。加快改善消费条件，推进苏宁广场、家乐福、沃尔玛等商业综合体开发，加快建设华东物料城、义乌小商品城等大型专业市场，推动农村商业连锁经营和统一配送，优化城镇商业网点布局，深入实施万村千乡市场和社区商业双进工程。健全重要农产品生产、储备和风险补贴制度，保障市场供应。市级财政投入1000万元，实施新一轮菜篮子工程。

巩固提升外贸出口。启动十百千工程，培育出口超5000万美元企业10家、超500万美元企业100家、超100万美元企业1000家，完成出口33亿美元，增长25%。完善电子商务平台，加强外贸市场开拓，全市开展电子商务企业达到400家。新兴市场出口商品占比提高2个百分点。实施品牌战略，强化特色培育，加快建设5个机电产品出口基地、2个科技兴贸创新基地，推进木材等专业化交易市场建设。创建省级以上出口品牌2个。

加强经济运行服务。完善会办服务机制，密切监测分析经济运行，有效协调要素供给，积极帮助基层和企业化解生产运营难题。强化项目建设服务，完善协调督办机制，加强立项审批、土地供应、资金保障等方面的跟踪协调，支持引导企业在结构调整、自主创新、节能减排等方面发挥主体作用，保障重点项目建设顺利推进。加强劳动力供需衔接，努力缓解结构性用工矛盾。完善中小企业服务体系，落实中小企业发展扶持政策，深入开展多形式的政银企对接，新增中小企业贷款200亿元。

二、大力推进重点突破，不断扩大沿海开发成效

强化政策推动，完善基础配套，加快实施沿海开发重点项目，扎实推进港口、园区、产业发展。

加速组合大港建设。全面推进30万吨级航道一期工程，加快浚深连云港区25万吨级航道，建成徐圩港区5万吨级航道主体工程，开工建设防波堤，灌河港区2万吨级航道建成通航。启动建设大堤作业区一期、墟沟作业区3个泊位和赣榆港区码头工程，建成主体港区氧化铝、散化肥泊位，积极推进30万吨原油码头、徐圩港区散货泊位等项目。南疏港公路、东疏港公路全线通车，北疏港公路建设加快推进，建设矿石码头皮带机输送系统，实现港区铁路电气化。完成港口基础设施投资30亿元。

加固基础设施支撑。加快推进310、204、226、242等国省道和临海高等级公路、新浦至徐圩公路建设，推进区域路网互联互通。加快实施连盐铁路工程，力争徐连客运专线开工建设。推进盐河航道整治，建设板浦内河港、中云台作业区二期和新安港区码头工程。完成交通建设投资40亿元。建成新沭河治理主体工程，实施蔷薇河下游出口段治理、徐圩新区输水支线及一批闸桥工程，开工建设东温庄水库。实施220千伏输变电工程5项，推进智能电网试点。

加快新型临港产业园区打造。强力推进徐圩新区开发，全面开工建设区内供水、热电、排污等配套基础设施，加快进口资源深加工和重大科研成果转化基地建设，建成产业服务中心和云湖商务服务区主体工程，完成基本建设投资100亿元。加快市开发区临港产业区项目集聚，提高投资密度和投入产出效率，提升柘汪、板桥、燕尾、灌河等沿海临港产业区开发水平，培育做大主导产业，形成一批以装备制造、新型化工为主导的特色产业园区。

加强政策和重大项目争取。推动国家东中西区域合作示范区获批建设，申报设立中日韩自由贸易区先导区和保税港区。加快推进江苏沿海开发银行总部和国家开发银行分支机构组建工作。强化重大项目对接，推动精品钢铁基地、炼化一体化、两翼港区和航空口岸开放、区域性大型机场建设列入国家十二五规划或专项规划。完成连淮铁路前期工作，争取整体煤气化联合循环发电、国电大型火电等项目尽早具备建设条件。

三、调整优化经济结构，构建富有竞争力的现代产业体系

实施新兴产业培育、临港产业集聚、服务业攀升和现代农业拓展工程，促进产业体系优化，增强产业竞争力。

致力培育战略性新兴产业。出台加快新兴产业发展意见，政策优先扶持、资金优先安排、技术人才优先引进，着力培育新医药、新材料、新能源、新型装备、新兴海洋、新型临港服务业等战略性新兴产业。推动市开发区五园五中心做强做优。推进建设新材料产业基地、高性能纤维及复合材料产业化基地、新医药产业基地、新能源产业和中小企业创新成果转化示范园，发展特色新兴产业园10家，加快培育创新药物、清洁能源、风电装备、高端硅材料、高性能纤维等创新集群。高新技术产业产值达750亿元，占规模以上工业比重达32%。

强力集聚两基型临港产业。依托港口、岸线、临港产业园区优势，积极引进发展基础性产业、基地型企业，加快百亿企业、千亿产业打造。新增年销售额过30亿元企业5家、过50亿元企业3家。开工建设田湾核电扩建、新海电厂超超临界机组等一批重大项目，加快推进甲醇制烯烃、镍合金制造、益海化工、德邦联碱、新能源汽车、航天光伏基地、亚邦产业园、索普工业园、博世汽车测试中心等一批重大项目建设，建成投产新海石化三期、金桥盐化氯碱等项目。造船、冶金、石化产业产值增长均超过40%。加快工业化与信息化融合，推动产业链向研发设计、营销服务等高端攀升，提升机械、纺织、化工、食品等传统产业发展水平，增强市场竞争力。

着力提速发展现代服务业。重点发展现代物流、研发设计、商务服务、文化创意、科技、信息等生产性服务业，加快发展生活性服务业，服务业增加值增长14%以上。启动建设航运交易市场，建成口岸一站式服务中心，增辟内外贸航线12条，打造金港湾国际物流园等一批物流平台，完成港口吞吐量1.5亿吨，集装箱运量450万标箱。民航客运量超过50万人次。加强10家重点服务业集聚区培育，抓好42个现代服务业重点项目建设。实施旅游项目深度开发，启动国家AAAAA级景区创建，加快邮轮经济等新业态发展，游客接待量、旅游收入分别增长15%、20%。大力发展文化产业，出台振兴文化产业政策，突出规划引领，坚持项目带动，强化市场运作，促进文化创意、文化博览、动漫游戏等产业发展。推进文化创意产业园、动漫产业园、西游记文化产业园、二郎神文化遗迹公园等一批文化产业重点项目建设，加快广播、影视、新闻出版等传统文化产业改造升级和做强做大，努力推动文化产业成长为支柱产业。

大力提升现代农业。开展高效设施农业、畜禽规模养殖和特色水产示范点创建，推进食用菌、鲜切花等五大特色产业板块做大做强。新增高效农业面积30万亩，设施农业面积15万亩，发展高效渔业10万亩、工厂化养殖5万平方米，畜禽规模养殖比重提高5个百分点。加强现代农业园区、农产品加工集中区、农产品市场体系等载体建设，加快生态观光农业、城郊都市休闲农业开发。新建市级以上现代农业示范区16家，发展农产品加工企业100家，增加省级以上龙头企业5家，农产品出口额增长10%以上。新增农民专业合作组织200个、土地流转面积20万亩。强化农产品质量安全建设，新增无公害、绿色和有机农产品品牌100个。稳定粮食生产。落实各项惠农政策，促进农民增收。

四、积极推动创新发展，加快推进转型升级

坚持以科技为引擎、人才为关键、教育为基础，推进创新驱动战略实施，大力培育创新型经济，建设创新型城市。

重抓创新能力建设。编制创新型城市建设总体规划，鼓励引导创新领域投入多元化，进一步提高全社会研发投入占地区生产总值比重。健全政府、企业、高校及科研院所共同参与的创新体系，建设淮海工学院、河海大学科技园和中国矿业大学、东北大学、南京理工大学、苏州大学等高校科技研发机构。建成市科技创业服务中心，新建企业院士工作站、工程技术研究中心等研发机构30个。新增科技孵化面积5万平方米。强化重大创新平台功能，加快中科院能源动力研究中心、康缘国家重点实验室、3个检测中心、6家高校研究院等研发平台建设，推进重大科技成果转化项目10项。加强创新型企业建设，培育省级以上创新型企业10家，支持企业主导和参与制订国际标准、国家标准和行业标准，争创自主知识产权和自主品牌。专利申请量、授权量分别达到1800件和1000件。

重抓人才引进开发。整合优化人才事业投入，促进政府、社会、单位和个人共同投资人才发展。继续实施高层次创新创业人才引进、高技能人才培养、海外人才集聚等工程，统筹推进各类人才队伍建设，引进本科以上学历人才4000人，培养高技能人才5000人。探索更加开放灵活的柔性流动方式，健全以能力业绩为导向的评价体系，完善激励保障创新创业、创造创优的人才机制，建立省级留学人员创业园1家。安排人才安置房100套。注重发挥项目引才、产业聚才作用，完成引智项目14项。

重抓教育基础支撑。充分发挥教育在引领创新发展、科学发展中的基础性作用。推动高等教育规模发展，引进南京医科大学等国内高校来连合作办学，促进在连高校提升办学层次，提高高等教育毛入学率。促进职业教育优化发展，加快打造一批重点专业群和实训基地，提高省级以上示范性职业学校办学水平。推动基础教育均衡发展，促进基本公共教育服务均等化，加快实施中小学校舍安全工程，开工建设市区义务教育学校3所，小学、初中入学率均达100%，高中阶段毛入学率96%以上。推进学前教育健康发展，学前三年入园率95%以上。

重抓资源环境保护。多措并举扎实抓好节能减排工作，严格行业项目准入，实行合同能源管理，狠抓重点领域节能，实施重点节能项目30项。建设柘汪临港产业区等污水处理厂3座，完善重点企业废水处理设施，完成省定节能减排目标任务。大力发展绿色经济、循环经济，加快低碳技术研发应用，积极创建省级循环经济示范工程，推进低碳园区、低碳企业试点工作，开展通榆运河水污染防治，实施清洁生产审核企业15家。进一步加大耕地资源保护力度，实施新一轮土地利用总体规划，提高土地集约节约水平，推进万顷良田建设工程。开展节水型城市创建，推广使用节能节材节水设备技术和材料，建成一批绿色工程、绿色建筑。完善海域使用管理，保护海洋生态环境。启动生态市建设，新增绿化造林面积12万亩，森林覆盖率达到23.3%。

五、着力深化改革开放，不断增强经济社会发展动力

围绕形成有利于科学发展、跨越发展的体制机制，加大改革攻坚力度，扩大开放合作，进一步激发经济社会发展动力与活力。

深化重点领域和关键环节改革。积极推进财税价格体制改革，完善预算编制和执行管理。创新投融资体制，规范国有资产经营平台运营，积极培育资本市场，大力发展股权、风险和创业投资，引进各类金融机构10家以上，推动3家企业上市或发行债券。探索资源性产品价格和环保收费改革。加快文化体制机制改革创新。抓好医疗卫生体制改革，推进公立医院改革试点，健全基本药物制度，政府办基层医疗卫生机构基本药物制度实施率100%。加快小城镇改革创新，探索新型基层经济管理体制，提升城镇社会建设水平。推进乡镇机构改革等农村综合配套改革，完善农村土地流转、征收征用制度，保障农民合法权益。

提升对外开放和合作水平。把招商引资与创新转型结合起来，推动外资投向战略性新兴产业、现代服务业等领域。推进产业链招商，优化产业生态，推动单一项目集聚向产业链集聚转变。加强专业化招商队伍建设，推进重大项目招引，抓好65个投资3000万美元或5亿元以上重点项目。强化服务推动，促进百家重点外资企业增资扩股。引进注册外资实际到帐12亿美元，内联客方到位资金480亿元。引导各类园区强化功能开发，建立投融资、技术研发、科技创业等生产性服务平台。加快日本生态产业园、深圳新型产业园建设。争取3家开发区升格为省级开发区，新增南北共建园区1家。加强与霍尔果斯等陆桥沿线口岸协作，做好对口合作支援工作，增开阿拉山口至连云港五定班列，增设内陆无水港2个。健全外派劳务管理机制，新增获得对外劳务合作经营权企业3家。

六、加快新型城镇化进程，统筹推进城乡一体化发展

坚持统筹城乡发展，推进新型城镇化建设，实施城乡建设项目1200个，完成投入450亿元，城市化率提高2个百分点。

强力推进国际性海滨城市建设。重点实施五大工程：一是新城开发拓展工程。全面加快连云新城建设，推进19条道路和14座桥梁工程，完成投资38亿元。加快实施科教创业园60项重点项目，完成投资40亿元，全面建成体育中心，3所中高职学校建成使用。加快孔望山新城、凤凰新城开发进程。中心城市建成区面积超过130平方公里。二是老城拆迁改造工程。加快推进新海城区西南出入口景观改造和墟沟老城区整治改造，实施锦屏磷矿、金桥盐化棚户区和南极北路、猴嘴片区等旧城改造，完成拆迁改造面积100万平方米。三是海滨特色打造工程。强化规划引领，加快推进花果山大道城市主轴功能带、山海景观轴、滨海休闲带开发，开工建设金海滩项目，建成城市之眼主体工程，完成中央商务区整治出新，建设商检大厦等项目。加大连岛开发力度，改造大路口中心街区等服务设施。四是市政设施配套工程。建设徐圩水厂一期工程等供水项目，新增城市自来水管道40公里、污水管网15公里。发展管道天然气用户1万户、住宅集中供热面积50万平方米，新增人防工程15万平方米。完善城市干线路网，实施市政路桥工程55项，修建便民道路30条，整治人行道板2万平方米。新辟优化公交线路5条，新增更新公交车50辆。五是人居环境改善工程。加快推进四城同创，实施玉带河景观带、猴嘴公园、街头绿地游园等一批绿化工程，新增城市绿地300公顷，城市绿化覆盖率达41.8%，申办省园博会，通过国家园林城市验收。着力提高居住满意度，推进省级无障碍城市建设，创建优秀物业管理小区15个。加强市容环境整治，抓好市区机动车停放设施建设和规范管理，新增一批环卫设施，促进集贸市场优化布点、提档升级，改善重点场所、行业卫生条件和秩序面貌。推进蔷薇河饮用水源地综合整治，饮用水源达标率100%，开展市区河流污染治理，改善水质。加强大气环境治理，控制机动车尾气和城市扬尘污染，空气质量优于国家创模标准。

加快构建新型城镇体系。大力推进四县县城建设，加快新区开发，完善承载服务功能。加强小城镇建设，提高温泉镇、杨集镇等6个重点中心镇、特色小城镇规划建设水平，新增市级城市化示范镇10个，加快形成规模适度、布局合理、环境整洁、各具特色的新型小城镇体系。四县完成城镇建设投资220亿元。扎实兴办农村实事，建成农村公路200公里，完成228条县乡河道疏浚、1900个河塘整治和19个乡镇农田水利设施集中连片治理，整治119个村庄环境，农村饮水安全工程增加受益人口15万人，新增无害化卫生厕所3万座。开展百村示范工程，推进康居示范村、农民居住点规划建设，创建省、市级康居示范村34个。继续实施脱贫攻坚，力争在全市基本消除绝对贫困现象。

七、坚持保障和改善民生，促进社会和谐进步

顺应人民群众过上更加美好生活的新期待，推动居民收入增长，健全社会保障，完善公共服务，不断提高城乡群众生活水平。

落实更加积极的就业政策。把促进充分就业作为经济社会发展优先目标，统筹推进城乡就业，多渠道开发就业岗位，新增农村劳动力转移3.5万人、城镇就业4.8万人。积极推动创业带动就业，实施小企业成长工程，新增私营企业5000家、注册个体工商户2万户。健全创业就业公共服务体系，加强技能培训，完成职业培训25万人次、创业培训3000人次、实用技能培训3.4万人次、农民培训12万人次。促进居民收入与经济增长协调发展，调整最低工资标准，市区、四县最低工资标准分别提高150元、130元，指导企业在岗职工工资适度合理增长，全市企业退休人员养老金标准人均提高10%。

进一步健全社会保障。完善社会保险、社会救助、社会福利和慈善事业相衔接的社会保障体系。不断扩大养老保险覆盖面，企业基本养老保险覆盖率达96%，新农保参保率达98%。加快城乡一体化医疗保险制度建设，城镇居民医保参保率达96%，新农合参合率稳定在96%以上，住院实际补偿比超过45%。推动城乡养老服务事业多元化发展，开工建设市残疾人集中托养机构。加强以最低生活保障为核心的社会救助体系建设，开展农村儿童先天性心脏病、白血病患者医疗救治工作，免费为500名贫困白内障患者开展复明手术。加快保障性安居住房建设，新建经济适用房3710套、廉租住房777套，增加公共租赁住房3280套，发放廉租房租赁补贴2500户，人均居住建筑面积15平方米以下城市低收入住房困难家庭实现应保尽保。

促进社会事业协调发展。优化城乡卫生资源配置，健全基层医疗卫生服务体系，促进基本公共卫生服务均等化，推进优质医疗资源集团化运作，组建市一院集团等3家医疗集团，建设市公共卫生中心，建成市东方医院病房楼。加快医疗救治、疾病控制和卫生监督体系建设，50%县区卫生监督机构通过省级考核评估。实施先进文化引领、历史文化传承、文化设施完善、文化精品打造和文化惠民利民五大工程，健全文化服务体系，提高公共文化产品和服务的供给能力。推进农村有线数字电视转换和影视文化城建设，建成开放市革命纪念馆，加快建设市老干部活动中心，启动市工人文化宫建设。加强基层文化发展，深入开展广场文化、惠民文化活动。贯彻落实《全民健身条例》，组团参加第七届全国城市运动会，广泛开展群众体育活动，推行医保健身一卡通，常年参加体育活动人群保持在50%以上。构建大人口服务管理机制，人口自然增长率控制在7‰。重视加强气象、防震减灾、地方志、外事、侨务、对台工作，推动妇女儿童、红十字、关心下一代事业再上新台阶。

重视加强社会管理。把稳定价格总水平放在更加突出的位置，加强价格调控监管，规范市场秩序，落实低收入群体价格上涨动态补贴制度，保障好困难群众基本生活。强化食品药品安全监管，完善质量安全检验检测机制，保障群众饮食用药安全。实施安全生产标准化建设，坚决遏制重特大事故发生。加强应急管理工作，提高突发事件应急处置能力。广泛开展群众性精神文明创建，提高居民素质和道德水平，推进社会信用制度建设，健全社会信用体系。建立新型社区管理和服务体制，深化村民自治、社区共治，强化村务公开民主管理。加强国防教育和动员，争创全国双拥模范城市六连冠。做好民族宗教工作。

切实维护和谐稳定。推进法治连云港、平安连云港建设，启动六五普法工作，强化社会治安综合治理，严厉打击各类违法犯罪活动，不断提升人民群众安全感。完善维权保障机制，加强协调劳动关系三方机制建设，构建和谐劳动关系。健全社会稳定风险评估和大调解机制，强化法律援助和服务，社会矛盾纠纷调处成功率达96%以上。高度重视信访工作，落实领导干部接访包案和信访代理制度，有效维护社会和谐稳定。

各位代表，实现十二五规划的目标愿景、完成开局之年的工作任务，对政府自身建设提出了新的更高要求。我们将进一步强化宗旨意识，规范行政行为，增强执行效力，提高服务效能，努力建设人民满意政府。一是坚持服务为民，转变职能。创新行政服务机制，深化政务公开，大力推进行政权力网上公开透明运行，做好政府新闻发布工作。整合12345政府服务热线和数字化城管平台资源，建成运行应急便民服务指挥中心。密切联系群众，更加注重倾听群众呼声、了解群众意愿，扎实办理市长信箱，认真解决群众反映强烈的突出问题。二是坚持执行为要，重抓落实。健全抓落实工作机制，加强重点工作督查考核，认真落实行政问责制。以分解细化政府工作报告目标任务为抓手，加强绩效管理。建立重大行政决策风险评估和实施情况后评价制度，进一步规范行政执法行为。自觉接受人大法律监督、工作监督和政协民主监督，扎实办理人大代表议案、建议和政协委员提案，主动听取各民主党派、工商联和人民团体意见建议，开展行政机关选聘民主党派、无党派人士和专家学者担任特约人员工作。三是坚持效率为先，提升效能。进一步规范和减少审批许可事项，提高行政审批效率，大力推进电子政务建设，完成市县乡三级联网，逐步实现网上电子审批。深化机关软环境建设，改进机关作风，严格控制会议、文件、庆典和评比达标表彰活动，创造良好服务环境。提升行政机关工作人员履职水平，增强服务发展本领。四是坚持廉洁为本，从严治政。认真落实党风廉政建设责任制，加强反腐倡廉制度建设。完善重大事项集体决策、专家咨询、社会公示等制度，加大执法监察、效能监察、廉政监察和审计监督力度，深入开展纠风整纪专项治理。坚持厉行节约，反对铺张浪费，继续对公务接待、公车使用、因公出国出境经费实行量化指标控制，有效压缩行政运行成本，努力保持为民务实清廉的良好形象。

各位代表，连云港的发展正在迎来一个新的五年黄金期。让我们紧密团结在以胡锦涛同志为总书记的党中央周围，深入贯彻落实科学发展观，在市委的领导下，意气风发、激情拼搏，锐意进取、扎实工作，为夺取全面建成小康社会新胜利、打造幸福美好新港城而努力奋斗！
